# Nonimmune Cells Contribute to Crosstalk between Immune Cells and Inflammatory Mediators in the Innate Response to *Trypanosoma cruzi* Infection

**DOI:** 10.1155/2012/737324

**Published:** 2011-08-18

**Authors:** Maria Pilar Aoki, Eugenio Antonio Carrera-Silva, Henar Cuervo, Manuel Fresno, Núria Gironès, Susana Gea

**Affiliations:** ^1^Departamento de Bioquímica Clínica, Centro de Investigaciones en Bioquímica Clínica e Inmunología (CIBICI), Consejo Nacional de Investigaciones Científicas y Técnicas (CONICET), Facultad de Ciencias Químicas, Universidad Nacional de Córdoba, Córdoba 5000, Argentina; ^2^Department of Immunobiology, School of Medicine, Yale University, New Haven, CT 06520, USA; ^3^Centro de Biología Molecular Severo Ochoa, Consejo Superior de Investigaciones Científicas (CSIC), Universidad Autónoma de Madrid (UAM), Cantoblanco, Madrid 28049, Spain

## Abstract

Chagas myocarditis, which is caused by infection with the intracellular parasite *Trypanosoma cruzi*, remains the major infectious heart disease worldwide. Innate recognition through toll-like receptors (TLRs) on immune cells has not only been revealed to be critical for defense against *T. cruzi* but has also been involved in triggering the pathology. Subsequent studies revealed that this parasite activates nucleotide-binding oligomerization domain- (NOD-)like receptors and several particular transcription factors in TLR-independent manner. In addition to professional immune cells, *T. cruzi* infects and resides in different parenchyma cells. The innate receptors in nonimmune target tissues could also have an impact on host response. Thus, the outcome of the myocarditis or the inflamed liver relies on an intricate network of inflammatory mediators and signals given by immune and nonimmune cells. In this paper, we discuss the evidence of innate immunity to the parasite developed by the host, with emphasis on the crosstalk between immune and nonimmune cell responses.

## 1. Introduction

The intracellular protozoan parasite* Trypanosoma cruzi* is the causative agent of Chagas disease, which is a health threat for an estimated 10 million people, living mostly in Latin America. More than 25 million people are at risk of the disease. It is estimated that in 2008 Chagas disease killed more than 10.000 people [1]. Although this infection occurs mainly in Latin America, in the past decades it has been increasingly detected in the United States of America, Canada, many European, and some Western Pacific countries. This is now a new worldwide challenge to nonendemic countries [1, 2]. The infective trypomastigote form invades macrophages and other cell types, where it is converted into the amastigote form and replicates. Acute manifestations often include parasitemia, which decays with the onset of immunity. Progression from the acute to the chronic phase coincides with the clearance of parasites from the blood stream and tissues. After years or even decades of primary infection, up to 30% of chronically infected people develop cardiac alterations, and up to 10% develop digestive, neurological, or mixed alterations [1].

Despite nearly a century of research, the most intriguing challenge for understanding the pathophysiology of Chagas' heart disease still lies in the complex host-parasite interrelationship. Different mechanisms have been defined to explain the pathogenesis of human and experimental Chagas disease. Among the mechanisms described, autoimmunity is the one that has received the most experimental evidence but also controversy [3–6]. Nevertheless, there are studies suggesting that parasite persistence in the host tissues is relevant in the pathogenesis of the disease [7–9]. Both theories recognize the transcendental role of innate immunity during host defense as well as in the development and progression of myocarditis during Chagas disease. Geographical variation in the severity of different forms of the disease indicates the importance of *T. cruzi* genetic variation in addition to host genetic background. Unfortunately, it remains as a neglected disease in the world, and, despite considerable research, effective vaccines and adequate drugs for *T. cruzi* infection are still lacking. In this paper, we discuss the evidence of innate immunity to the parasite developed by the host, with emphasis on the crosstalk between immune and nonimmune cell responses, and its role in sustaining defense as well as injurious processes.

## 2. Role of Toll-Like Receptors in the Innate Immune Recognition of *Trypanosoma cruzi*

The innate immune response is initiated by pattern-recognition receptors (PRRs), which recognizes pathogen-associated molecular patterns [10, 11]. Different PRRs generally recognize diverse ligand specificities. The broad specificities of the PRRs and their ability to form functional multireceptor complexes allow large combinatorial repertoires. This further diversifies the recognition and signaling of cooperating PRRs and enables the host to detect almost any type of pathogen, discriminate between different microorganisms, and mount a competent immune response. The PRRs most widely investigated are the toll-like receptors (TLRs). This receptor family comprises 10 and 13 functional members in humans and mice, respectively. Besides sensing pathogens, ranging from bacteria to fungi, parasites, and viruses, it is now thought that they recognize endogenous ligands which have an important role in the regulation of inflammation as well as in noninfectious disease [11]. Studies focusing on host innate immunity against *T. cruzi* infection demonstrated that these receptors are crucial for many aspects of microbial elimination, including recruitment of phagocytes to infected tissues and subsequent killing [10–12]. However, it has been reported that, activated to excess, TLRs can mediate pathology [12]. The TLR signaling pathways consist of two cascades: a myeloid differentiation primary-response-gene-88- (MyD88-) dependent pathway and a Toll/IL1R-domain containing adaptor protein inducing IFN*β* (TRIF-) dependent (MyD88-independent) pathway. The MyD88-dependent pathway mediates the production of proinflammatory cytokines through all TLRs except for TLR3, while the TRIF-dependent way is indispensable for the induction of type I IFNs through TLR3 and TLR4 [13]. 

Taking into account that proinflammatory cytokines produced by TLR activation play an important role in the immunopathology of chronic Chagas' cardiopathy, it has been proposed that a single-nucleotide polymorphism in the genes that encode proteins in TLR signaling could play an important role in differential susceptibility to Chagas disease. Thus, it was recently demonstrated that *T. cruzi*-infected individuals who are heterozygous for the MAL/TIRAP S180L variant lead to a decrease in signal transduction upon ligation of TLR2 or TLR4 to their respective ligands, which is associated with lower risk of developing chronic Chagas' cardiomyopathy [14]. 

**Figure 1:**
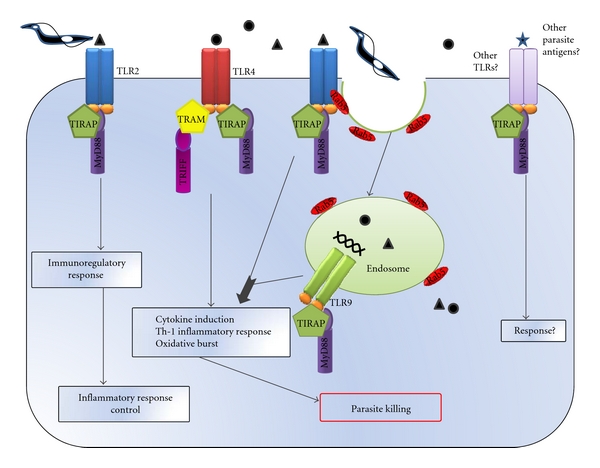
TLR-dependent innate immune responses. *T. cruzi*-derived components are recognized by TLR2, TLR4, and TLR9, triggering the production of proinflammatory cytokines and microbicidal effectors. Thus, parasite antigens and the cytokines locally released act together to promote the development of protective Th1 response, which lead to parasite growth control. Moreover, TLR2 signaling also has immunoregulatory properties essential to hinder the immune response induced by the parasite. Regarding the *T. cruzi* entry process, TLR2 but not TLR4 is involved in the activation of Rab-5, fusion of early endosomes, and phagocytosis of trypomastigotes. Furthermore, it is plausible to think that others unexplored TLRs and/or parasite antigens could be involved in the induction of the innate immune responses against *T. cruzi. *

TLRs are expressed on different immune cell populations, including macrophages, dendritic cells (DCs), B lymphocytes, specific T-cell subsets, and even on nonimmune cells such as fibroblasts, parenchyma cells, and epithelial cells. The importance of TLRs during *T. cruzi* immune response was initially evidenced by studies performed with professional antigen-presenting cells [15], in which the authors remark the importance of TLR2 as a mediator of the defense mechanisms during the early stages of the host response to infection. Internalization of intracellular parasites by phagocytosis is a key event in the initiation of the immune response, with phagosomal maturation being central to microbial killing and antigen presentation. Regarding the *T. cruzi* entry process, several studies have examined the mechanisms of invasion or internalization of this parasite, being the host cells and the host molecules involved in this interaction still not completely understood. Interestingly, we have recently demonstrated that activation of small guanine-phosphonucleotide-binding proteins Ras-related protein- (Rab-) 5, fusion of early endosomes, and phagocytosis induced by trypomastigotes in macrophages, involved TLR2 but were independent of TLR4 [16] (Figure 1). Signaling through the TLR2 by the parasite-released-antigen Tc52 stimulated the maturation of DCs and strikingly rescued immunized mice from lethal infection [17]. Moreover, the activation of TLR2 leaded to the secretion of chemokines inducing leukocyte recruitment [18]. Thus, parasite antigens and the cytokines locally released may act together to promote DC maturation and subsequent development of protective Th1 response. In our lab we also found that the inoculation of TLR2-synthetic ligand prior to infection *in vivo* improved the survival of lethally infected mice [19]. Noticeably, other authors showed that infected TLR2(−/−) mice produced enhanced levels of cytokines suggesting that*, in vivo*, TLR2 may have a predominant immunoregulatory role during acute infection with *T. cruzi *parasites, at least with the Y strain [20]. However, these authors observed no major difference in parasitemia and mortality between infected TLR2 knockout and wild-type mice. Furthermore, MyD88 knockout mice were more susceptible to *T. cruzi*, with higher parasitemia and greater mortality [21]. Additional studies attributed most of the MyD88-dependent host resistance to the cooperative activation of TLR2 and TLR9 [20] (Figure 1). The activation of TLR9 by *T. cruzi* came from early studies showing that parasite genomic DNA stimulates cytokine responses in professional presenting cells [22].

The study of linkage between *T. cruzi* innate immunity and the generation of adaptive immune response has been scarcely explored. Recently, it was proposed that a weak TLRs activation might contribute to the relatively slow expansion despite strong CD8+ T cell response during acute *T. cruzi* infection. This study was performed evaluating the frequency of parasite-specific CD8 T cells among other parameters. The authors found an earlier but transient induction of this cell population by the administration of the combination of TLR9 plus TLR2 agonist concomitantly with the infection [24]. Otherwise, Oliveira and colleagues (2010) found that *T. cruzi*-infected TLR2(−/−), TLR4(−/−), TLR9(−/−) or Myd88(−/−) mice generated both specific cytotoxic responses and IFN*γ*-secreting CD8+ T cells at levels comparable to wild-type mice, although the frequency of IFN*γ*+ CD4+ cells was diminished in infected knockout Myd88 mice [25]. Thus, the authors concluded that neither the lack of each TLR2, TLR4, or TLR9 nor the absence of all MyD88-mediated pathways affect the development of cytotoxic function and number of CD8+ T cells, which are crucial effectors against this parasite.

The potent immune response elicited by *T. cruzi* requires the generation of immunoregulatory network in order to prevent or minimize reactivity to selfantigens or an excessive response to the parasite. It has become clear that active suppression mediated by regulatory T-cell (Treg) populations is crucial for the control of the immune response both in human and experimental *T. cruzi* infection [26, 27]. It has been demonstrated that Tregs display an increased level of TLR2, TLR4, TLR5, TLR7/8, and TLR10 expression compared to conventional effector CD4+ CD25− T cells, suggesting that the expansion and function of this regulatory cells may be closely influenced by TLR ligands [28–31]. In line with this, the immunoregulatory role for TLR2 reported during the acute infection could be explained by the fact that the suppressive function of Tregs is directly controlled by the triggering of TLR2 but not TLR4 or TLR9 [32]. TLR2 ligands activate the expansion of Tregs by an indirect effect via antigen-presenting cells or by direct TLR2 triggering of Tregs. Moreover, signaling through TLR2 strongly enhance CD25 expression inducing an increased sensitivity to interleukin (IL) 2. It is believed that the increase in IL2 receptor expression on Tregs [32] and IL2 production by effector T cells temporally abrogate the suppressive capacity of the Tregs *in vivo* [31] (Figure 1). Therefore, it is plausible to think that TLR2 ligands provided by the parasite could first expand Tregs and abrogate their suppressive phenotype. When low numbers of the pathogen are present, as in the persistent phase of infection, Tregs regain their immune-suppressive phenotype and could be responsible for the pathogen persistence.

## 3. TLR Ligands from *Trypanosoma cruzi*

*T. cruzi* display numerous ligands for the TLRs. In 2001, Gazzinelli's team found two potent TLR2 activators; the protozoan trypomastigote surface-highly purified glycosylphosphatidylinositol (GPI) anchors linked to the surface mucin-like glycoproteins, and free GPI anchors named glycoinositolphospholipids (GIPLs) were recognized through TLR2. These parasite ligands trigger IL12, TNF*α*, and nitric oxide (NO) production by inflammatory macrophages [15, 33]. Regarding other parasite molecules, it was reported that the *T. cruzi* Tc52-released protein induces human DC maturation signaling through TLR2. Tc52 comprises two homologous domains, which contain a glutathione-binding site and a hydrophobic C-terminal region, and is essential for parasite survival and virulence. Authors proposed that Tc52 would be one candidate molecule to design a multicomponent vaccine to control *T. cruzi* infection [17]. On the other hand, Oliveira et al. (2004) observed that *T. cruzi*-derived GIPL ceramide, in high concentration, could activate mouse cells through TLR4 *in vitro.* Furthermore, TLR4-mutated C3H/HeJ mice were highly susceptible to *T. cruzi* infection [34]. 

In addition, Bafica and colleagues demonstrated that *T. cruzi*-DNA, a TLR9 agonist, stimulated cytokine production by antigen-presenting cells and cooperatively participated in the control of infection [20]. A more recent study identified the ODNs containing CpG motifs in the *T. cruzi* genome responsible for the immunostimulatory activation of TLR9 from mouse and human infected cells, suggesting that the killing of parasites may be required to release agonists of TLR9 [35]. Remarkably, infected double knockout TLR2(−/−)TLR9(−/−) mice developed a parasitemia equivalent to animals lacking MyD88 but did not show the mortality displayed by MyD88(−/−) animals. Authors suggest that TLR9 has a primary role in the MyD88-dependent induction of IL12/IFN*γ* synthesis during infection.

Summing up, although some parasite ligands have been reported as TLR agonists, it is plausible to think that other molecular patterns from this complex parasite may activate different combination of TLRs on target/effector cells. The combined activation of these receptors would drive the final outcome of host cellular response determining the defense as well as tissue damage. 

## 4. Toll-Like Receptor-Independent Innate Immune Responses to *Trypanosoma cruzi* Infection

As was discussed above, it is well established that the TLR-dependent pathway initiates an effective innate immune responses against *T. cruzi*. However, infection of cells deficient for expression of the TLR adaptor proteins TRIF and MyD88 still produces cytokines in response to this protozoan, suggesting that other TLR-independent pathways also may be activated during the early immune response. In this sense, new families of PRRs have emerged as important components of the innate immune system that sense the presence of this microorganism and drive the host defense to a protective phenotype. 

**Figure 2:**
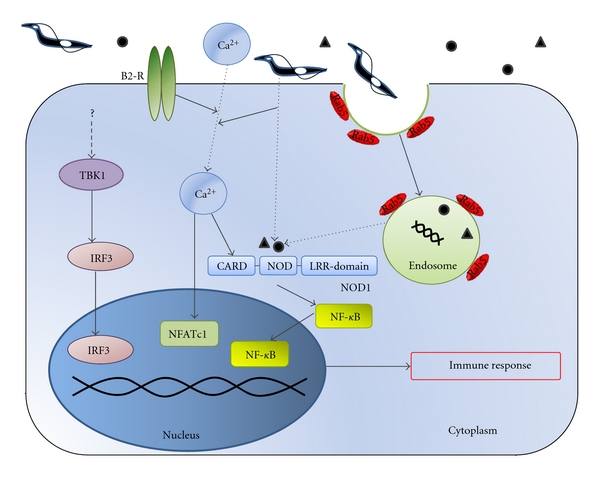
TLR-independent innate immune responses. Infection triggers increased intracellular Ca^2+^ concentration through interaction of bradykinin with the bradykinin B2 receptor (B2-R) among other mechanisms. Target innate immune cells utilize Ca^2+^ to activate the Ca-dependent signaling pathway leading to the activation of NFATc1. Intracellular *T. cruzi* is recognized by NOD1, activating NF-*κ*B. *T. cruzi* is also recognized by unknown molecules leading to the activation of TBK1 and IRF3. Altogether the mechanisms participate in the induction of an effective immune response against the parasite.

The NOD-like receptors (NLRs) comprise a large family of intracellular PRRs responsible for the recognition of microorganisms independent of TLR signaling [36]. The first and better characterized members of this family are NOD1 and NOD2 [37, 38]. Although these receptors were extensively characterized as PRRs for bacterial and viral infection, little is known regarding their role in the recognition of intracellular parasites, that is, *T. cruzi*. In this regard, Silva and coworkers (2010) recently demonstrated that the effective response required for host resistance to infection was exclusively mediated by NOD1 but not by NOD2 receptor [39]. Despite normal cytokine production in the sera, NOD1(−/−) mice were highly susceptible to this infection, as judged by the high parasite load in spleen and heart tissues and succumbed to the infection in a similar way to Myd88 and nitric oxide synthase (iNOS) knock-out mice. In light of their results, the authors concluded that the NOD1-dependent response may be implicated in host resistance to *T. cruzi *by mechanisms independent of cytokine production (Figure 2).

Strikingly, *T. cruzi* infection is able to activate other innate immune pathways in the absence of TLR signaling, although the sensing molecules that recognize the parasite ligands are still unknown. Studies performed *in vitro* showed that trypomastigotes trigger IFN*β* expression in immune and nonimmune cells by engaging a novel TLR-independent pathway that requires both TANK-binding kinase 1 (TBK1) and IFN-regulatory factors (IRF)3 [40] (Figure 2). Although the role of IFN*β* in the protection against parasite infection remains controversial [41, 42], it was demonstrated that IFN*β* is responsible for resistance of macrophages infected with *T. cruzi* mainly in the absence of MyD88 [43].

Furthermore, the activation of one member of the nuclear factor of activated T-cell (NFAT) family transcription factors NFATc1 mediated IFN*γ* production by macrophages and DC, developing an effective Th1 response and DC maturation during *T. cruzi* infection in double-knockout mice (Myd88−/− and Trif−/−), despite high sensitivity to the infection [44]. A pivotal signaling for the activation of NFATc1 is mediated by the Ca^2+^ pathway. Previously, it was demonstrated that the parasite increases intracellular Ca^2+^ through interaction of kinins (bradykinin) with the bradykinin B2 receptor, which is another defense mechanism (Figure 2). In infected tissues, trypomastigotes induce a robust secretion of chemokines and plasma extravasations in macrophages via TLR2, thus providing the substrates for the proteolytic generation of kinins, which are also involved in DC maturation and IL12 production [45–48]. 

## 5. Innate Immune Response in Nonimmune Target Tissues Elicited By *Trypanosoma cruzi*

It is known that innate immune cells, including macrophages and DCs, play pivotal roles in immune response; however, nonimmune cells such as parenchyma cells, epithelial cells, endothelial cells, and fibroblasts, among others, also contribute to immunity development [49]. Thus, the outcome of the immune response in a target tissue depends not exclusively on the immune cells but also on the intricate network and signals given by immune and nonimmune cells. Furthermore, although the dominant feature of the innate immune system is to protect the host from infectious agents, it may have other roles in mammalian biology. For example, TLRs on parenchyma cells have been demonstrated to be involved in tissue repair and homeostasis [50, 51].

Accumulative evidence demonstrates that the liver has specific immunological properties and contains a large number of resident and nonresident cells that participate in the regulation of inflammatory and immune responses [52, 53]. Although Kupffer cells are considered the primary cells to respond to pathogen-associated molecular patterns, recent studies provide evidence that multiple populations of nonhematopoietic liver cells, including sinusoidal, endothelial cells, stellate cells, and hepatocytes, express and respond to PRR signaling as well as taking on the roles of antigen-presenting cells [52–54].

Liver cells express a variety of TLRs, which have been shown to participate in hepatic tissue injury and repair, and contribute to the pathogenesis of a variety of liver diseases [52, 55]. However, the action of TLRs on liver cells in host defense against invading pathogens is less clear. The liver is the target of a wide range of microbes including *Listeria*, *Salmonella,* and *Plasmodium* species. However, there are few data related to the implication of *T. cruzi* experimental infection and the relevance of the innate immune response against this parasite in this organ [56, 57].

**Figure 3:**
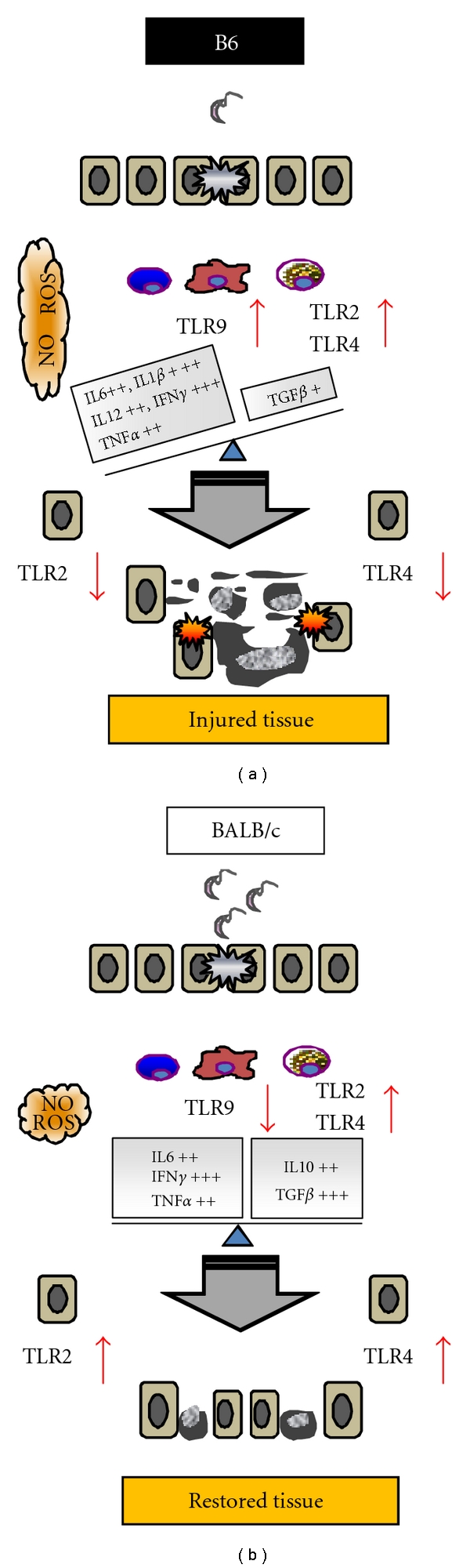
Comparative analysis of hepatic injury, inflammation and TLR expression in *Trypanosoma cruzi-*infected B6 and BALB/c mice. The parasitemia was higher in BALB/c than B6 mice. However infected B6 mouse strain showed stronger and injurious inflammatory environment (increased NO and ROS) associated with high levels of TLR2, TLR4, and TLR9 in hepatic leukocytes. In contrast, BALB/c mice displayed more balanced proinflammatory/immunoregulatory cytokines profile during the acute infection. Furthermore, TLR2 and TLR4 were upregulated in infiltrating leukocytes and hepatocytes as well, while TLR9 expression was low in hepatic leukocytes of infected BALB/c mice. Altogether the results suggested that the strong inflammatory environment elicited in infected B6 mice plus the loss of TLR2 signaling may be responsible for the severity of the hepatic injury and higher mortality of this mouse strain [19, 23].

We have reported a severe hepatic injury in B6 mice infected with Tulahuen *T. cruzi* trypomastigotes. We noted that this mouse strain showed a higher mortality than BALB/c mice, associated with an unbalanced proinflammatory cytokine profile, a decreased TLR2 and TLR4, and an increased TLR9 expression in liver [23]. Supporting our results, it was demonstrated that *T. cruzi*-infected TLR2 knockout mice produced higher levels of proinflammatory cytokines and NO than wild-type mice. These results suggest that TLR2 has an important immunoregulatory role preventing excessive activation of innate immunity and uncontrolled production of proinflammatory cytokines [33]. Furthermore, we also showed that infected BALB/c mice developed a softer environment where the balance between cytokine storm and immunomodulatory signaling given by TLR2 and TGF*β* may modulate the inflammatory damage in the liver [19] (Figure 3). We additionally demonstrated a stronger expression of hepatic iNOS and a higher NO production by liver leukocytes of infected B6 compared to BALB/c mice [19]. Several authors have described that reactive oxygen species (ROS) can induce cell death by either apoptosis or necrosis in liver pathologies [58, 59]. In this sense, an enhanced and sustained nicotinamide adenine dinucleotide phosphate (NADPH) oxidase p47-phox expression and the coexpression of gp91 and p47-phox were found only in liver from infected B6 [19]. Thus, the activation of NADPH oxidase enzymatic complex would be a key player in the liver damage, probably as an instrument contributing to liver apoptosis and necrosis during infection in B6 mice (Figure 3). In addition, we found that while TLR2 and TLR4 expression on hepatic immune infiltrating cells was similar in both mouse strains, TLR9 expression showed a clear difference in hepatic leukocytes. Thus, only leukocytes from infected B6 mice sustained high expression of TLR9 throughout the acute phase. These results support the hypothesis that continuous TLR9 signaling might contribute to excessive and harmful inflammatory response in infected B6 mice. In accordance with our results, a crucial role of TLR9 during *T. cruzi *infection was shown [20]. Interestingly, in hepatocytes we found that TLR2 and TLR4 are differentially modulated in infected BALB/c and B6 mice, suggesting that these innate immune receptors would play a role not only in immune cells but also in liver parenchyma cells (Figure 3). In this sense, it has been postulated that TLR signaling in parenchyma cells would be a key mechanism to prevent death caused by excessive cytokine release [60, 61]. 

There are increased evidences demonstrating the potential role of TLR-ligands treatment as therapeutic approach and they have shown to be highly effective in the protection against protozoan, among them *T. cruzi* [14, 39, 40]. In our study we further observed that pretreatment with Pam3CSK4, a TLR2/TLR1 agonist, before infection induced a marked reduction of proinflammatory cytokines, nitrite, and transaminase levels and a decrease in the number of hepatic inflammatory foci and consequently in the mortality of infected mice [19]. In this study we postulate that the inadequate integration of signals involving molecular (TLRs, cytokines, NO, and ROS) and cellular (immune and parenchyma cells) components influences the outcome of local immune response during this parasite infection. Moreover, the differential TLR and cytokine modulation in the liver, induced by *T. cruzi* infection, emphasize the importance of local innate immune response in hosts with different genetic background and could contribute to the understanding and the design of novel immune strategies in controlling liver pathologies.

On the other hand, local innate immunity also has a key role in the pathophysiology of several cardiovascular diseases. The heart muscle, initially thought to be a bystander in the immune response to *T. cruzi*, has been found to be an active participant in the innate response, a hypothesis firstly postulated by Postan et al. (1999) [62]. During this infection, cardiomyocytes are actively integrated in the inflammatory response releasing NO, cytokines, and chemokines which, in turn, attract leukocytes to the inflammatory site and control intracellular parasite replication [63–67]. Cardiac cell exposure to proinflammatory cytokines may pre-condition the myocardium environment to temporarily protect cardiomyocytes from growth factor deprivation-induced apoptosis [68]. In fact, we found that *T. cruzi* infection protects isolated cardiac myocytes from apoptotic cell death induced by serum deprivation, and this effect was due to an increase in Bcl-2 molecule. Interestingly, we also found that the infected cardiomyocyte culture pretreated with inactive cruzipain, a major parasite antigen, enhances antiapoptotic protection as well [69, 70]. In a recent study, we explored the nature of the crosstalk between cardiac innate immunity and *T. cruzi* infection. We found that the triggering of TLR2 signaling could be playing an important role in cardiomyocyte protection elicited by *T. cruzi *(Ponce et al., results submitted). Another study indicates that signaling through TLR2 and NF-*κ*B activation also led to the production of IL1*β*, which mediated the cardiomyocyte hypertrophy observed in Chagas' myocarditis [71].

Adipose tissue has also emerged as an important target for infection, since a significant number of parasites are found within this tissue during the chronic phase of infection [72]. Because the adipocyte act as an active endocrine cell, it is plausible to speculate that these cells may be critically involved in the progression and reactivation of the disease. Adipose tissue contains a number of different cell types. A massive macrophage (F4/80-positive cells) influx was observed in adipose tissue during acute infection. Thus, macrophages and adipocytes combined may be important contributors to systemic inflammation. In adipose tissue, TNF*α*, IFN*γ*, and IL1*β* protein expression were upregulated at least 10-fold compared with noninfected mice. *In vitro* studies with a cell line model for adipocytes (3T3-L1) revealed that the levels of TLR2 and TLR9 but not TLR4 expression were upregulated. In addition, IFN*γ*, TNF*α*, and IL1*β* were also increased under infection [73]. 

Taken together, the results cited here make it clear that TLR signaling contributes in the beginning and development of the immune response, but the resolution does not depend on individual pathways but on the integration of multiple signals. The combined activation of different PRRs can result in complementary, synergistic, or antagonistic effects that modulate innate and adaptive immunity [11].

## 6. Microbicidal Activity of Effector Cells and Inflammatory Mediators against *Trypanosoma cruzi*

Arginine and tryptophan metabolism in macrophages depends on cytokine-inducible enzymes and produce mediators involved in microbicidal or suppressive mechanisms in the context of infection. Classical activation of macrophages by Th1 cytokines during infection by intracellular parasites is thought to be protective, whereas alternative activation by Th2 cytokines is involved in the survival of extracellular parasites. Thus, iNOS and arginase have been involved in the regulation of the Th1/Th2 balance during immune processes, and have been used as markers for M1/M2 activation, respectively [74].

Arginase and iNOS metabolize L-arginine, a semi-essential amino acid, to L-citrulline plus NO and urea plus L-ornithine, respectively. Two arginase isoforms have been described in mammals encoded by different genes [75]. Arginase I is cytoplasmic and is highly expressed in liver and alternatively activated macrophages by Th2 cytokines (IL4, IL13) [76, 77] and also by IL10, TGF*β*, GM-CSF, and prostaglandin E_2_ (PGE_2_) [76, 78, 79]. Arginase II is mitochondrial and expressed in a wide variety of tissues and cell types, mainly in kidney [80] and cardiomyocytes [69], and is induced by TLR ligands [81]. The product of arginase activity, L-ornithine, can be metabolized by ornithine aminotransferase giving L-proline, which is required for collagen synthesis and by ornithine decarboxylase (ODC), which results in polyamine synthesis needed for proliferation of all eukaryotic cells. 

There are three NOS isoforms: neural NOS (nNOS), endothelial NOS (eNOS), and iNOS, which catalyze the oxidation of L-arginine to L-citrulline and NO. Activated iNOS is found in a diversity of cell types in the immune system [82] and also in cardiomyocytes [83]. The most common inducer for iNOS is IFN*γ* combined with LPS, but other cytokines such as IL12, IL1*β*, and TNF*α* are also able to induce it. 

*In vitro T. cruzi* infection triggers the induction of potent NO-dependent trypanocidal activity in infected cardiomyocytes [65] and macrophages [84–89]. In addition, the interaction of macrophages with apoptotic cells through vitronectin receptor increases TGF*β* and PGE_2_ release, which promoted parasite proliferation by increasing ODC activity [90]. 

The *in vivo* role of iNOS in *T. cruzi* infection is still debated, because experiments with iNOS-deficient mice are contradictory [91–93]. However, the administration of iNOS inhibitors to infected mice results in increased parasitemia and mortality, indicating a protective role [94]. On the other hand, when excessive, NO can also have a cytotoxic effect in the host and lead to immune suppression of T cells. In addition, NO production during acute *T. cruzi* infection in rats was inhibited in peripheral blood monocytes, due to the increase of arginase activity [95]. In the mouse model of acute infection, it has been described that the expression of both arginase isoforms and ODC is higher in susceptible BALB/c mice than in C57BL/6 mice [96]. This was associated with an increased parasite burden in BALB/c heart tissue. Interestingly, arginase II was expressed by cardiomyocytes, whereas arginase I was found in infiltrating CD68^+^ macrophages. These results suggest that infection induces arginase expression, which may not only influence host cell and parasite survival but which might also downregulate the counterproductive effects triggered by iNOS in the heart during infection. The myeloid-derived suppressor cells (MDSCs), which increase during acute *T. cruzi* infection, also express iNOS and arginase, and they are highly efficient in suppressing activated T cells [95]. It is possible that the induction of iNOS and arginase seen in infected hearts suppresses T-cell activation, allowing parasite replication. In this direction, it is possible that arginase-expressing infiltrated macrophages are MDSCs. 

Moreover, the immunization of susceptible BALB/c mice with cruzipain resulted in enhanced anti-inflammatory cytokine secretion, associated with the induction of a CD11b^+^ GR1^+^ spleen immature myeloid population that exhibited arginase, but not iNOS, activity [97]. This phenotype is compatible with the MDSC population. Furthermore, cruzipain-stimulated naive macrophages released IL10 and TGF*β* and displayed enhanced arginase activity, favoring *T. cruzi* growth [98, 99]. By contrast, the immunization of resistant C57BL/6 mice with cruzipain resulted in the secretion of IL12 and IFN*γ* and consequently the induction of iNOS messenger and protein expressions as well as high NO production [100]. These findings point up the importance of host genetic background in macrophage response. In another study, macrophages from mice, immunized with a plasmid DNA containing the gene encoding the catalytic domain of *T. cruzi* transsialidase, were able to effectively kill intracellular parasites by a NO-dependent mechanism [101]. Furthermore, CD4 and CD8 T-cell clones are able to produce IFN*γ* that inhibits parasite replication into macrophages. These results encourage the use of this strategy for developing vaccines against Chagas disease [102].

The inflammatory cytokines also induce the enzyme indoleamine-pyrrole 2,3-dioxygenase (IDO) in macrophages, which converts the essential amino acid L-tryptophan to N-formylkynurenine. During *T. cruzi* infection, there is a systemic activation of IDO, and its inhibition induces an exacerbated parasite load and infection-associated pathology. Further, the authors demonstrated that treatment of *T. cruzi*-infected mice with the IDO downstream metabolite, L-kynurenine, was able to kill the parasite and to improve the survival of lethally infected mice. Moreover, IDO activity was critical to control *in vitro* parasite's replication despite the high production of NO produced by IDO-blocked *T. cruzi*-infected macrophages [103]. In summary, IDO activation and a high iNOS/arginase balance are related to a better outcome of the disease. These evidences suggest that intervention of IDO and iNOS/arginase pathways could be useful in antitrypanosomatid therapeutic strategies for acute infection.

The production of superoxide anion (O2−) by neutrophils and other phagocytes is an important event in innate immune response. This metabolite is the precursor of a range of chemicals referred to as reactive oxygen species. Although these act as microbicidal agents and kill invading microorganisms, there is growing evidence to suggest that myocardium from patients with Chagas disease is exposed to sustained oxidative stress-induced injuries involved in disease progression [104]. The superoxide anion is mainly produced by the multiprotein enzyme complex NADPH oxidase, which is inactive in resting phagocytes but becomes activated after interaction with pathogens and their subsequent engulfment in the phagosome [105]. In response to a pathogen stimulus, the soluble subunits p47phox, p67phox, and p40phox translocate en bloc to the membrane, where they bind flavocytochrome b558. It is clear that inflammatory cytokines are key players in the induction of the oxidative metabolism. Macrophages exposed to IFN*γ* and TNF*α* became primed to a state of enhanced responsiveness by the respiratory burst with the induction of membrane and cytosolic components. During *T. cruzi* infection, neutrophils, murine splenocytes [106, 107], blood monocytes, and macrophages produced ROS and destroyed intracellular forms of this parasite [108, 109]. However, ROS are also produced by infected cardiomyocytes, and signal the production of proinflammatory cytokines through the activation of NF-*κ*B, thereby contributing to maintaining the sustained inflammatory state observed in Chagas disease [110].

In our laboratory, we recently demonstrated that cruzipain was able to induce ROS production by splenocytes and macrophage line RAW 264.7. This parasite glycoprotein triggered NADPH oxidase activation and induced the production of several ROS *in vitro*, mainly O2− [111]. As expected, macrophages, derived from cruzipain-immune mice, primed *in vivo* with IFN*γ* and TNF*α*, produced more ROS than naive macrophages. This work was the first to report that oxidative stress can be induced by a *T. cruzi* antigen. 

It has been proposed that strong oxidants, macrophage-derived peroxynitrites (ONOO^−^), arising from the reaction of NO with superoxide radical (O2−) participate in cytotoxic mechanisms against *T. cruzi* inside the phagosome. More recently, it was demonstrated that internalization of trypomastigotes by macrophages triggers ONOO^−^ formation when NO and O2− were produced simultaneously intraphagosomally. This microbicidal mechanism was evidenced by amastigote killing, detected by nitroxidative protein modifications and parasite ultrastructural alterations [112].

Summing up, NO, ROS, and additionally ONOO^−^ [113, 114] are also very efficient mechanisms in the fight against pathogens. However, these reactive oxygen and nitrogen species are very cytotoxic and, when excessive, can result in tissue damage and promote inflammatory diseases.

## 7. Concluding Remarks

In this paper we emphasized the importance of the TLR signaling pathway in the innate immune response to the protozoan parasite *T. cruzi*. This parasite has multiple ligands that elicit a potent innate immunity and the subsequent development of adaptive immune response. This activation pathway leads to pro- and antiinflammatory cytokine synthesis. While there is much evidence indicating that MyD88 is a crucial molecule for activation of this type of receptor, other TLR-independent mechanisms in host-parasite interaction are being elucidated. Thus, it has been demonstrated that NLRs which recognize pathogens in the cytoplasm are involved in parasite recognition. Furthermore, several other mechanisms that induce intracellular Ca^2+^ influx as well as activation of NFATc1 and bradykinin B2 receptor can be activated by this parasite infection. The combined activation of TLRs and other cytoplasmic receptors opens new and interesting viewpoints in our understanding of the synergistic or antagonistic combined action of different PRRs.

The knowledge of the role of TLRs in the pathogenesis of Chagas disease and the identification of new *T. cruzi*-derived TLR ligands is not only important for developing better adjuvant to be used in vaccines, but also new immunotherapy to prevent or minimize Chagas disease pathology. In addition, new pharmacological drugs that disrupt TLR signaling may be attractive when excessive pathology-associated inflammation occurs, as well as in experimental acute infection.

## Abbreviations

DC: dendritic cell
GIPLs: glycoinositolphospholipids
GPI: glycosylphosphatidylinositol
IDO: indoleamine-pyrrole 2,3-dioxygenase
IL: interleukin
IRF: IFN-regulatory factors
MDSCs: myeloid-derived suppressor cells
MyD88: myeloid differentiation primary-response gene 88
NADPH: nicotinamide adenine dinucleotide phosphate
NFAT: nuclear factor of activated T-cell transcription factors
NLRs: NOD-like receptors
eNOS: endothelial nitric oxide synthase
iNOS: inducible nitric oxide synthase
nNOS: neural nitric oxide synthase
NO: nitric oxide
NOD: nucleotide-binding oligomerization domain
ODC: ornithine decarboxylase
ODNs: oligodeoxynucleotides
Pam3CSK4: tripalmitoylcysteinylseryl-(lysyl-) 4; TLR2 ligand
PGE2: prostaglandin E2
PRRs: pattern recognition receptors
Rab: Ras-related protein 5
ROS: reactive oxygen species
TBK1: TANk-binding kinase 1
TIRAP/MAL: TIR domain that contains the adaptor protein, also known as MAL
TLRs: toll-like receptors
Treg: regulatory T cell
TRIF: Toll/IL1R-domain containing adaptor protein-inducing IFN*β*.
